# The pandemic experience survey II: A second corpus of subjective reports of life under social restrictions during COVID-19 in the UK, Japan, and Mexico

**DOI:** 10.3389/fpubh.2022.913096

**Published:** 2022-08-24

**Authors:** Mark M. James, Jamila Rodrigues, Morgan Montoya, Natalia Koshkina, Federico Sangati, Ekaterina Sangati, Matthew Ratcliffe, Havi Carel, Tom Froese

**Affiliations:** ^1^Embodied Cognitive Science Unit, Okinawa Institute of Science and Technology Graduate University, Onna, Japan; ^2^Department of Philosophy, University of York, York, United Kingdom; ^3^Department of Philosophy, University of Bristol, Bristol, United Kingdom

**Keywords:** survey, COVID-19, cross-cultural, longitudinal, phenomenology, lived experience, social distancing

## Background

In August 2021, Froese et al. published survey data collected from 2,543 respondents on their subjective experiences living under imposed social distancing measures during COVID-19 (1). The questionnaire was issued to respondents in the UK, Japan, and Mexico. By combining the authors' expertise in phenomenological philosophy, phenomenological psychopathology, and enactive cognitive science, the questions were carefully phrased to prompt reports that would be useful to phenomenological investigation and theorizing (2–4). These questions reflected the various author's research interests (e.g., technology, grief, time).

Between April 7th and July 31st, 2021, a second questionnaire with the same question set was issued to respondents of the original who had agreed to do a follow-up. This was intended to capture subjective reports of life under social distancing measures a year after the initial survey. By this time–depending on their country of residence and health status–respondents had potentially lived with repeated and prolonged lockdowns and a variety of other restrictions on their social lives.

When taken together, Survey I and Survey II provide a cross-cultural and longitudinal dataset that allows for analysis of longer-term impacts of imposed social distancing measures on people's experiences. For researchers working in diverse disciplines, this dataset offers a rich resource that reflects people's reactions to the imposition of different social restrictions in different countries and over different time periods.

Another motivation for this work is to contribute to efforts to keep historical records of the COVID-19 pandemic (5). Our contribution includes detailed accounts of how people experienced various dimensions of the pandemic from their first-person perspective.

To contextualize participants' responses, we provide a brief overview of the pandemic situation in each country throughout the data collection period. Crucially, it is not our intention to draw any meaningful correlations between the figures and insights enumerated below and our survey data. We offer them to help elucidate some of the general conditions within which the subjective reports were made. We focus only on some key details derived from data made available by OurWorldinData.com (6).

There was much heterogeneity in policy responses to managing the virus during this period, so general claims are not warranted. However, figures from the COVID-19 Stringency Index compiled by OurWorldinData.com indicate that the severity of measures–a composite of nine response indicators including school and workplace closures, travel bans, and restrictions on public gatherings–fluctuated in all regions during the collection period, but with an overall decrease in the UK, as opposed to increases in Japan and Mexico. Beginning April 2021, out of a possible 100 (100 indicating the most severe measures), the UK was rated at 70, Japan at 42, and Mexico at 47; by the end of July 2021, the numbers were 44, 50, and 67, respectively.

For any given individual, innumerable variables impact their particular experience. However, some core variables may be assumed to inform the general tone throughout the collection period: case numbers, death rates, and vaccine implementations. Of course, other variables will have been broadly significant too, such as access to testing, prevalence of facemasks and familiarity with wearing them. But in the interest of space, we limit our discussion to the core variables mentioned.

This period saw increases in daily case numbers for each region. Mexico started in April 2021 with 41 new daily cases per million people and was at 144 by the end of July. The UK began April at 66 and finished July at 378, with a peak during that period of 794 on July 17th. Japan saw 21 new cases on April 1st, rising to 99 by the end of July.

Deaths during the same period, according to Ourworldindata.com were relatively low and stable in each region. Mexico hovered around 3 deaths per day per million people throughout; the UK around 1, and Japan 0. It is worth noting that different countries had different testing regimes and criteria for counting COVID-19 deaths which makes any final international comparisons on such matters difficult.

Vaccines were becoming more available during this time, but their distribution was still quite uneven. By the end of July, the share of people who completed the initial vaccine protocol was 56% in the UK; 30% in Japan; and 20% in Mexico.

Finally, it is worth mentioning that since publishing our initial report (1), much research continues to highlight the widespread negative impacts of the pandemic on mental health, for example: Winter and Lavis (7), Chishima and Liu (8), Schafer et al. (9), Lantos et al. (10), Dettmann et al. (11), Loch et al. (12), and Pearce et al. (13). However, as was true at the time of our first report, the effects of this crisis have not been homogenous, with some positive changes also being noted (14–17). In order to better make sense of this diversity of findings, future efforts may be directed at merging insights derived from population-level assessments with analysis of individual-centric subjective reports. Rich evidence of these heterogenous trends is collected in the data accompanying this report.

## Methods

We ran the questionnaire on SurveyMonkey.com from April 7th to July 31st, 2021. In order to facilitate diachronic analyses, the questionnaire consisted of the same 42 questions as the first. Participants were recruited by email from the 1,036 who agreed during Survey I to participate in a follow-up. We collected 562 responses in total. Some did not meet the criteria for inclusion: 1 participant did not grant his/her consent to participate, 19 participants did not fill out their name and email address, and thus did not proceed to the rest of the survey. Five hundred and forty three participants fulfilled the criteria, and their responses are included in the corpus that we are hereby making openly available.

### Questionnaire

**Figure 1:**
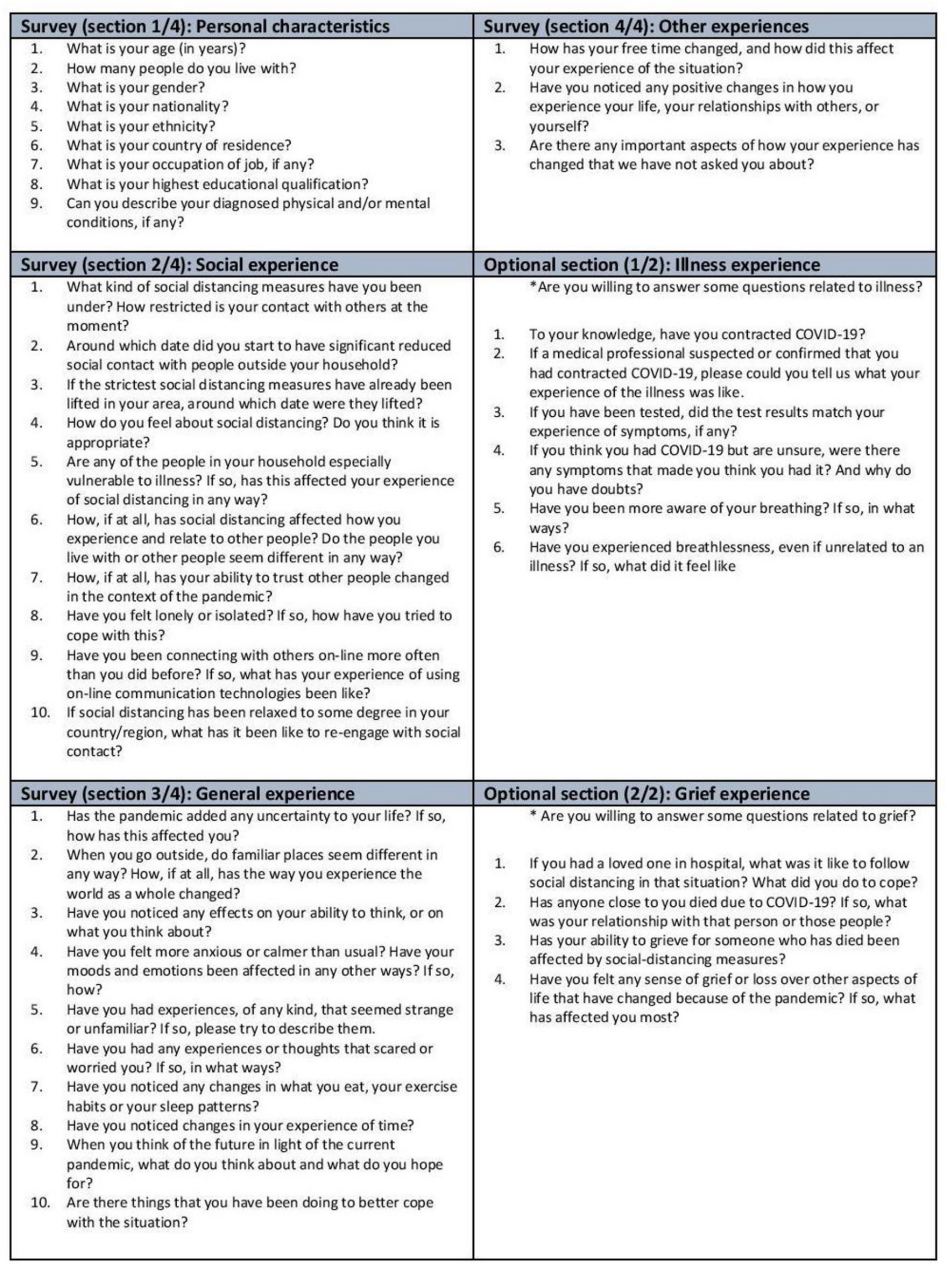
Questionnaire consisting of six sections highlighted in distinct colors with a total of 42 questions. The same set of questions was used in Survey I also (1). Section 1 (“Personal characteristics”) consisted of nine questions regarding the demographic data and self-described medical history of the participant. Section 2 (“Social experience”) consisted of eight open-ended questions about social experience and two questions regarding the date of lockdown measures. Section 3 (“General experience”) consisted of 10 open-ended questions about general experiences, such as the subjective experience of time and space and coping mechanisms. Section 4 (“Other experiences”) consisted of three questions about other experiences, like the occurrence of any positive changes and hopes for the future. Section 5 (“Illness experience”) consisted of six questions related to experiences of illness. Section 6 (“Grief experience”) consisted of four questions about the experience of grief.

The questionnaire consisted of six sections with 42 questions (see Figure 1 for details; the English version can be downloaded along with the corpus). Participants were free to answer as many questions as they wished, and were informed that Sections 5 and 6 dealt with more sensitive topics. These questions were only displayed if participants confirmed their willingness to participate.

### Data structure

The corpus comprises 5 comma-separated value (CSV) files (EN.csv, JP.csv, JP2EN.csv, ES.csv, ES2EN.csv), where each row contains the answers from a single participant and each column corresponds to a particular question. Each participant is identified by a unique ID that follows the convention LL_CC_DDDD (e.g., EN_UK_0379), where LL stands for language (EN, ES, JP), CC country of residence (UK, MX, JP, OO for other, NA not specified), and DDDD is an incremental number of the received response based on the starting timestamp.

To easily compare responses between the two questionnaires, we provide an additional CSV file with aligned answers (export_EN_sv1_sv2.csv) in which each cell contains answers from participants who took part in both questionnaires (prefixed with the string “V1” and “V2,” respectively). In this file, Spanish and Japanese answers are translated into English. Participants' responses to questionnaire 1 and 2 were aligned using email addresses. However, we could not align 4 participants due to differing emails. Therefore, this file includes answers from 539 of the 543 participants in the corpus mentioned above.

As with the previous report, given that participants self-selected and were free to answer as much or as little as preferred, we cannot exclude the possibility that this introduced biases into the corpus. Thus, caution should be exercised when interpreting the data.

### Qualitative and quantitative data comparison

One ambition in collecting this data is to apply both quantitative and qualitative approaches to its analysis. It is worth saying a few words about our methods as they might be instructive to others who wish to work with this data (see the Supplementary Information for details).

One challenge with having multiple large data sets of responses to open-ended questions is that it is slow to systematically analyse so many responses according to the standards of qualitative research. We address this challenge by adopting a mixed-methods approach. This includes quantitative sentiment analysis using Google Cloud Natural Language API (Application Programming Interface), qualitative coding analysis using Atlas.ti, and phenomenological interpretation of subjective reports.

Taking an illustrative example from one of our projects on the link between affectivity and technology during the pandemic, we first used the Google Cloud Natural Language API to automatically calculate sentiment scores for all participant responses. Having done that, we manually assessed the API's reliability and confirmed it to be valid within a desirable range. Responses were then sorted from most positive to most negative. This, in turn, allowed us to filter out 66% of responses (a reduction from 1,009 to 344), leaving only those with the most emotionally salient content for further qualitative analysis (18, 19). In sum, combining these methods allowed us to target relevant data and thus to expend our time and energy more efficiently.

## Description of corpus content

Of the 543 included respondents, 539 answered at least one open question. The word count for the entire English translated corpus is 209,019 words with 974,696 characters.

### Survey participants

The breakdown of respondents by language is: 401 English, 80 Spanish, and 62 Japanese respondents. Participants come from 31 different nationalities with ages spanning from 21 to 88. More detailed demographic data is found in Table 1.

**Table 1:** Statistics and demographics of the pandemic experience corpus from survey II.

	**English**		**Japanese**		**Spanish**		**Total**
Total responses	415	74%	64	11%	83	15%	562
Selected responses	401	74%	62	11%	80	15%	543
Responded to at least 1 open-ended question	399	74%	60	11%	80	15%	539
Word count	175,821		1,290		31,908		209,019
**Gender**
Male	98	63%	30	19%	28	18%	156
Female	293	78%	30	8%	51	14%	374
Other	4	100%	0	0%	0	0%	4
**Age**
Range	21–88		21–86		25–82		21–88
Mean	56		54		49		54
Median	59		54		51		57
**Country of residence**
UK	297	100%	0	0%	0	0%	297
Mexico	31	29%	0	0%	75	71%	106
Japan	16	21%	60	79%	0	0%	76
Other	54	92%	1	2%	4	7%	59
**Ethnicity**
White	341	97%	2	1%	10	3%	353
Hispanic	34	34%	0	0%	67	66%	101
Asian/Pacific Islander	6	9%	59	91%	0	0%	65
Black	0		0		0		0
American Indian or Alaskan Native	1	100%	0	0%	0	0%	1
Multiple/Other	16	84%	0	0%	3	16%	19
**Education (highest qualification)**
Primary education	1	50%	0	0%	1	50%	2
Junior High or equivalent	6	86%	1	14%	0	0%	7
High school	29	76%	5	13%	4	11%	38
Vocational training	36	78%	7	15%	3	7%	46
Bachelor or equivalent	125	66%	31	16%	34	18%	190
Master or equivalent	134	78%	6	4%	31	18%	171
Doctoral	67	79%	11	13%	7	8%	85
**COVID-19**
Yes	13	54%	0	0%	11	46%	24
Suspected	31	89%	0	0%	4	11%	35
Not sure	40	66%	13	21%	8	13%	61
No	258	78%	29	9%	44	13%	331

There are a total of 30 different countries of residence, with the majority of the participants residing in the UK, Japan, and Mexico. Fifty nine participants are living outside these three areas. We expect this latter figure is a reflection of how the addresses targeted in Facebook are not always aligned with addresses in the real world. The corpus follows a similar gender distribution to the Survey I: English-, Japanese-, and Spanish-language respondents self-identified as “female” 74, 50, and 65%, respectively. Crucially, some ethnicities, such as “Black” or “American Indian,” are entirely or almost entirely absent from this data. Consequently, the included data should not be understood as broadly representative.

### Main themes

We outline some themes that stood after a preliminary analysis, making some comparisons between data from Surveys I and II. Under each theme heading we include some illustrative examples, along with participant IDs. Where possible, we use examples that contrast responses from particular individuals across both questionnaires. Of course, by the time data for Survey II was being collected vaccines had already begun rolling out and different variants of the virus had been circulating widely. As such, it is likely that closer comparison of the data between corpora will reveal much insight about the impact of such variables on experience, but this is not the place for that detailed analysis.

## COVID-19 infections

In Corpus I, there were 9 participants who reported having contracted COVID-19 (*n* = 9), whereas in Corpus II there were 24 reports (*n* = 24). Participants in Corpus II described accompanying symptoms as mild, moderate, and severe. The most common symptoms described by participants were fever (EN_UK_0013), fatigue (EN_UK_0285), loss of smell and taste (EN_00_0382), headaches (EN_00_0053), flu-like symptoms (EN_MX_0325), and body pain (EN_MX_0474). However, alongside these relatively anticipated symptoms, some less common symptoms were also mentioned, including “COVID-toe” (EN_UK_0150; EN_UK_0024) and “long-term COVID” (EN_UK_0108).

## Bodily awareness

Bodily awareness is understood in this context as an awareness of physiological aspects and functions of the body. One participant displayed a change of diet between their two questionnaire responses. In Corpus I, they reported eating more sugary foods, while in Corpus II, they reported eating healthier meals to reduce weight, motivated by the link between obesity and poor COVID-19 outcomes (EN_UK_0519). Participants also reported awareness of potential physical symptoms and attendant bodily sensations related to COVID-19, e.g., breathing awareness (JP_JP_0031; ES_MX_0130), awareness of coughing (EN_UK_0029; JP_JP_0268; ES_MX_0508). Additionally, participants reported harm prevention strategies associated with such bodily events, e.g., temperature checking (EN_UK_0148; JP_JP_0081), more hand washing (ES_MX_0119), and disinfecting (ES_MX_0270).

## Digital communication

Participants continued to report using online communication tools in their daily lives. They reported that they worked from home (EN_UK_0042), studied online (EN_MX_0514), socialized with their friends and family (ES_MX_0387), and got physical exercise through digital means (EN_UK_0324). Preliminary analysis reveals that “online” was mentioned by 36% of UK participants (*n* = 107), preferred social media applications were mentioned by 17% (*n* = 51), and video-conferencing applications were mentioned by 55% (*n* = 164). Some respondents adapted to the increased need for digital communication and stopped bemoaning the lack of eye contact and body language between Corpus I and II (EN_UK_0152, ES_MX_0387). In contrast, some participants reported dissatisfaction in both corpus I and II due to the lack of body language (EN_UK_0418; EN_UK_0092). Other participants were tired of the dominance of online meetings (EN_UK_0042) or reported experiencing extremely negative emotions relating to video-conferencing software (EN_UK_0103).

Given that Q48 was the target of some more in-depth quantitative analysis for other projects (18, 19), we can briefly say what some of that revealed. Evaluating the general mood of all the Q48 answers across both Corpus I and II, we can state that the general mood in response to online communication technologies has become more negative. Within the aligned corpora, the number of respondents with a Clearly Negative valence increased by 25%, from 66 in Corpus I to 83 in Corpus II. This increase is despite the fact that the number of respondents who answered this question in Corpus II decreased by 8.7% (from 528 in Corpus I to 482).

## Time

There was a wide range of experiences of time for participants. One participant noted in Corpus I that while there were visual cues to the changing seasons, the days and weeks were indistinguishable; one “runs into another.” In Corpus II, the same participant notes that time was “doing 2 different things at the same time,” whereby the “lockdown seems forever” but the “year is flying by” (EN_UK_0282). In contrast to these disturbances, a few participants reported a sense of growing efficiency in their use of time (EN_UK_0025; EN_JP_0027). In short changes in how one sensed the speed of time were relatively common (e.g., EN_00_0038; EN_MX_0384, EN_UK_0240). Others reported more severe disruptions in their experience of time, e.g., forgetting the current year (EN_UK_0142), disturbance with their internal body clock and concept of time (EN_UK_0092). But there is great heterogeneity in people's responses to this question, with some participants reporting no changes in their experience of time at all (JP_JP_0207).

## Coping

Adaptive coping strategies include “positive thinking” (EN_UK_0076), change to a healthier diet and lifestyle (EN_UK_0019), more exercise (EN_UK_0105), connecting with nature (EN_UK_0173), yoga (EN_UK_0024), seeking spiritual guidance (ES_MX_0489), and maintaining interpersonal relationships (EN_UK_0010). One example of growth in coping abilities comes from EN_00_0082, where in Corpus I the participant reported engaging in casual activities such as reading or watching movies, in Corpus II the same participant is being much more active and intentional in their coping strategies, from meditating and playing sports to planning their daily actions and “building a better present” for themselves. Conversely, and consistent with Corpus I, we also find that others found it hard to maintain a positive attitude during the pandemic, sometimes adopting less adaptive coping strategies, like avoidance (EN_UK_0388), increased consumption of antidepressants (ES_MX_0305), and alcohol (EN_JP_0431).

### Trust

Corpus II shows that after a year into the pandemic, the topic of trust, which was already a central theme of Corpus I, continued to be highlighted by respondents. For example, in Corpus I, “less trust in government” was mentioned by 31% (*n* = 92) of the UK participants, 4% (*n* = 4) of the Mexico participants, and 8% (*n* = 6) of the Japan participants. In Corpus II, “less trust in government” was mentioned by 19% (*n* = 55) of the UK participants, 7% (*n* = 7) of the Mexico participants, and 5% (*n* = 4) of Japan participants. Between Corpus I and II, a few participants displayed a continuous lack of trust in government (EN_UK_0425) and acknowledged a continual decline in trust with friends (EN_00_0073). Lack of trust in health care practice was also discussed (EN_UK_0444). However, diminishing trust is not generalizable across all participants, as other individuals reported no change in trust with family and friends between Corpus I and II (EN_UK_0172; EN_UK_0348), and some reported growth of trust between the two time periods (JP_JP_0045).

## Grief

Corpus II contains many responses referring to bereavement over people who died whilst living under social restrictions. A recurring theme with such bereavement was the impact of social distancing measures on mourning. Some individuals report the isolation of experiencing the death of a loved one (ES_MX_0394; EN_UK_0079). One participant reported in Corpus II that grieving from afar felt like a “fictitious mourning” (ES_MX_0385). Many of such deaths were attributable to COVID-19 (e.g., ES_MX_0122). However, bereavement was not the only type of grief expressed by participants. Other types of grief were related to a broader range of losses, such as deteriorating relationships (EN_UK_0013), the loss of physical touch specific to hugging (EN_UK_0296; EN_UK_0337), the loss of freedom (EN_UK_0469), a loss of group participation, such as performing in an orchestra (EN_UK_0319).

We also observe changes in people's experiences of grief across the data sets. For instance, in Corpus I respondent EN_UK_0432 replies to Q78, which asks about having experiences of grief, “I'm sorry I cant think of any.” In Corpus II, however, this respondent writes, “Not being able to travel to see the loved ones is really hard. Im not light-minded anymore I miss being a nice easygoing person.” To the same question, EN_UK_0379 writes in Corpus I how they have experienced “loss related to not being able to move forward” with their plans, but that it was only “temporary.” In Corpus II, on the other hand, the respondent expresses a deepening sense of loss “over the wasted time, and the loss of the life” they “expected to have been able to build by now.”

## Outlook

In our previous report, we anticipated this second survey, suggesting that it would enable researchers to “perform longitudinal analyses of people's responses, comparing their experience after the initial wave of the pandemic with their experience after the onset of vaccination programs” (1). As was suggested in the background section, and as should be apparent from the above themes, there is much heterogeneity concerning the impacts of the pandemic on people's experiences. With the diachronic perspective on people's subjective experiences recorded in these two corpora, we are now well-positioned to look more closely at the conditions that undergird such heterogeneity. One might ask how specific living conditions and coping strategies bolster against the adverse effects of social isolation. Or, how, for instance, certain resources, skills and abilities enable the maintenance of communities of care, even when close physical connection is limited. One of the main challenges of the pandemic has been to develop individual and collective ways of flourishing despite the stress, uncertainty, and disruptions to our social lives. Many reports of such efforts and the conditions that facilitated them are captured herein. Perhaps future studies aimed at supporting human flourishing in adversity could be informed by this dataset.

## Data availability statement

The datasets presented in this study can be found in a Figshare online repository (doi: 10.6084/m9.figshare.20443182) at the following link: https://figshare.com/articles/dataset/The_Pandemic_Experience_DATE_RANGE_/20443182.

## Ethics statement

The studies involving human participants were reviewed and approved by Human Subjects Research Review Committee (HSRRC), Okinawa Institute of Science and Technology Graduate University (OIST). The patients/participants provided their written informed consent to participate in this study.

## Author contributions

TF and MR initiated the project. TF and JR conceived of conducting this follow-up study, which reissued questions to participants from a previous study (1). FS implemented the questionnaire as an online survey, managed participant recruitment, extracted the corpus, and performed initial natural language processing and statistical analyses. JR, MM, and MJ performed the initial data coding, while NK helped with follow-up coding and quantitative analysis. ES preformed the statistical analysis. MJ and MM wrote the first draft of the manuscript, which all authors helped review thereafter. All authors contributed to the article and approved the submitted version.

## Funding

This work was supported by JSPS Topic Setting Program to Advance Cutting Edge Humanities and Social Sciences Research (Grant Number JPJS00120350377).

## Conflict of interest

The authors declare that the research was conducted in the absence of any commercial or financial relationships that could be construed as a potential conflict of interest.

## Publisher's note

All claims expressed in this article are solely those of the authors and do not necessarily represent those of their affiliated organizations, or those of the publisher, the editors and the reviewers. Any product that may be evaluated in this article, or claim that may be made by its manufacturer, is not guaranteed or endorsed by the publisher.
