# Corrigendum: The pandemic experience survey II: A second corpus of subjective reports of life under social restrictions during COVID-19 in the UK, Japan, and Mexico

**DOI:** 10.3389/fpubh.2023.1141279

**Published:** 2023-01-24

**Authors:** Mark M. James, Jamila Rodrigues, Morgan Montoya, Natalia Koshkina, Federico Sangati, Ekaterina Sangati, Matthew Ratcliffe, Havi Carel, Tom Froese

**Affiliations:** ^1^Embodied Cognitive Science Unit, Okinawa Institute of Science and Technology Graduate University, Onna, Japan; ^2^Department of Philosophy, University of York, York, United Kingdom; ^3^Department of Philosophy, University of Bristol, Bristol, United Kingdom

**Keywords:** survey, COVID-19, cross-cultural, longitudinal, phenomenology, lived experience, social distancing

In the published article, there was an error in the article title. Instead of “**The pandemic experience survey II: A second corpus of subject reports of life under social restrictions during COVID-19 in the UK, Japan, and Mexico**”, it should be “**The pandemic experience survey II: A second corpus of subjective reports of life under social restrictions during COVID-19 in the UK, Japan, and Mexico”**

The authors apologize for this error and state that this does not change the scientific conclusions of the article in any way. The original article has been updated.

