# Supplementary material for: The pandemic experience survey II: A second corpus of subjective reports of life under social restrictions during COVID-19 in the UK, Japan, and Mexico
Source: Front Public Health. 2022 Aug 24;10:913096. doi: 10.3389/fpubh.2022.913096 (PMC9449414; doi:10.3389/fpubh.2022.913096)
Supplement: Supplementary file 1 [file Data_Sheet_1.docx]

Supplementary Materials

These supplementary materials provide additional details of some work we have already carried out on this data set, which was pointed towards in sections 2.3 and 3.2.3. It derives from addressing the challenge of large textual corpora analysis and the application of quantitative methods for the primary filtering of the data.

The biggest challenge with working on the collected dataset was due to its size. It was not feasible to systematically analyse all the responses according to the standards of qualitative research. Instead, we relied on the Google Cloud Natural Language API tool, which allowed us to apply automatic sentiment scoring to all responses in booth corpora, and then use that information to sort, filter, and find correlations for the further qualitative analysis of the sections that were expected to be of most interest.

In what follows, we will describe our methodology in more detail, focusing as an example on the analysis of responses to Question 48: *Have you been connecting with others on-line more often than you did before? If so, what has your experience of using on-line communication technologies been like?*

**Instrument verification**

At the outset, we had to test the reliability of the instrument ( i.e Google Cloud Natural Language API) that we were planning to use for performing the initial analysis, and to calibrate it to our set of records.

In the first stage of the validation process we ran all the responses through the API and got automatically assigned values of score and magnitude for each one. The score value can range from -1 to 1, where -1 corresponds to a Clearly Positive valence, 0 would be a Neutral, and 1 - Clearly Negative. The magnitude value indicates how emotionally charged the responses are. The magnitude ranges from 0 to infinity. **Table 1** shows how different score and magnitude value combinations ​​can be interpreted.

**Table 1**. Example values of sentiment score and magnitude and their interpretations

| **Valence** | **Score** | **Magnitude** |
| --- | --- | --- |
| Clearly Positive | 0.8 | 0.3 |
| Clearly Negative | -0.6 | 0.4 |
| Neutral | 0.1 | 0.0 |
| Mixed | 0.0 | 4.0 |

According to instructions provided by Google, score values close to 0 can be assigned both to a low-emotion text or to the one expressing contradictory emotions with both high positive and negative charges that cancel each other, thus resulting in a numerical indicator close to 0. In this case, the valence code of the response should be determined by assessing the combination of the numerical value of score and magnitude. A high magnitude value means a high emotional charge of the text and therefore indicates a Mixed, rather than Neutral, valence.

At the second stage of the validation process, we manually assigned qualitative valence codes to each response to Question 48, using four possible categories that correspond to the ones suggested by Google: Clearly Positive, Clearly Negative, Neutral, and Mixed.

We identified two features of the targeted question that led us to make two methodological assumptions. The first assumption was made because in reality we had to deal not with one question, but with two packed together. Answers to the first question (*Have you been connecting with others on-line more often than you did before?)* entailed a statement of fact whereas the answers to the second question (*If so, what has your experience of using on-line communication technologies been like?)*, entailed an emotional assessment of this fact and contained the sentiment we were actually interested in.

Since the majority of respondents answered *Yes* to the first part of the question, thereby giving a significant positive charge to the entire response, we assumed that this might introduce noise into our manual valence coding and tilt the total number of answers towards Clearly Positive. Therefore we agreed not to take this part of the response into account while manually coding the sentiment.

The second methodological assumption was made because some respondents, while making a fairly significant contribution, nevertheless did not address the actual question. This caused some confusion initially since we tried to assess the Valence in relation to the question posed. However, considering that the Google Cloud Natural Language API provided sentiment analysis regardless of the relevance of the response to the question posed, we decided not to take into account the relevance of the answer and evaluate only the emotional component.

The qualitative coding was done by two researchers independently of each other across 1009 answers of both Survey I and II. This independent verification resulted in 922 matching codes, which is 91.2% of the entire corpus. In order to make a more accurate assessment of interrater reliability, we calculated the value of Cohen's Kappa, which turned out to be 0.88. According to the recommendations of Landis and Koch (1977) for the interpretation of kappa values, this value was close to perfect, which allowed us to conclude that our manual coding was credible.

We then held an inter-rater discussion with a third researcher to reach a consensus on the valency codes for those 88 responses that the first two parties disagreed with. As a result, we compiled a corpus of responses for Question 48 with manually assigned valence codes and automatically assigned Score and Magnitude values.

To evaluate the accuracy of Google Cloud Natural Language API predictions, we looked at the distribution of Valence codes on Score and Magnitude scales. The first thing to draw attention to when analysing this distribution is that, as shown in **Figure 1**, we do not see any breakdown of Neutral and Mixed Valence codes on the scale of Magnitude. The main takeaway from this observation is that Magnitude is not informative for predicting Valence. Therefore, the findings that follow are based solely on the analysis of the distribution of valence codes ​​on the Score scale.

**Figure 1**

Distribution of Valence Codes on Score/Magnitude Chart


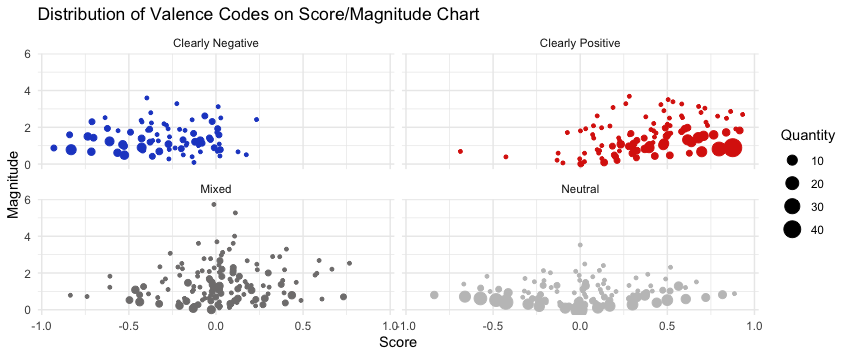


As seen in **Figure 2**, Clearly Negative coded responses gravitate toward negative score values, while Clearly Positive coded responses ​​gravitate towards positive Score values. However, we also observe some irregularities which do not correspond to expectations: three (2.0%) Clearly Negative coded responses fall into the positive part of the Score scale, and six (2.1%) Clearly Positive coded responses fall into the negative part. Furthermore, among Clearly Positive responses, we can see two outliers with the Score values -0.8 and -0.4. See **Table 2** below.

**Table 2.** Clearly Positive valenced responses assigned with negative score by Google Cloud Natural Language API. It is not clear to us why these examples were assigned with the scores they were by the API, and without additional investigation it is difficult to specualate as to what the precise causes may be. However, given the accuracy of the automatic scoring, as verified by our interrater coding, such outliers can safely be ignored.

| **Respondent** | **Answer** | **Valence code** | **Score** |
| --- | --- | --- | --- |
| EN_00_0073 | Online communication has been a gift really.. otherwise, those lock down days would...for me...have been intolerable.. | Clearly Positive | -0.8 |
| JP_JP_0562 | Even though I'm online, I feel that it is not much different from face-to-face contact. | Clearly Positive | -0.4 |

**Figure 2** Distribution of manually assigned Valence Codes along the Google Cloud NLP API Score Scale


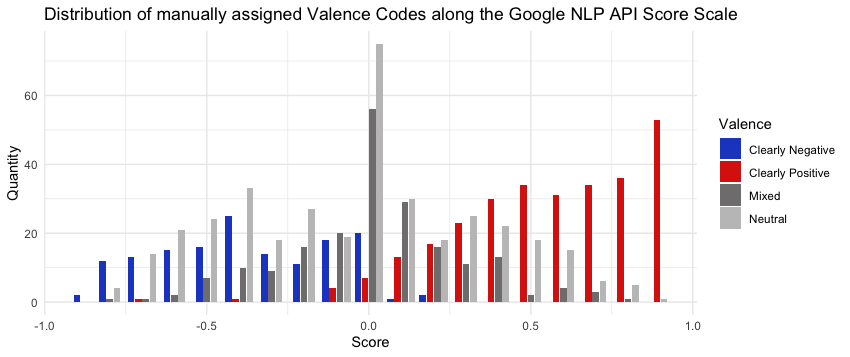


However, since the margin of error is so small, we can state that Google Cloud Natural Language API provides quite accurate predictions on valence code based on the score value and can be used to automatically identify Clearly Positive and Clearly Negative responses.

Furthermore, the calibration that Google calls for allowed us to completely get rid of irregularities (apart from the outliers) in the corpus. As shown in **Figure 3**, the intersection of Clearly Negative and Clearly Positive Valence codes occurs exclusively in the area of ​​the lower and upper whiskers. Therefore, we can cut off Clearly Negative valence codes by the value of the upper quartile (Q1=-0.1), and Clearly Positive valence codes by the value of the lower quartile (Q3=0.4).

**Figure 3**

Score, Median, Mean and Interquartile Range


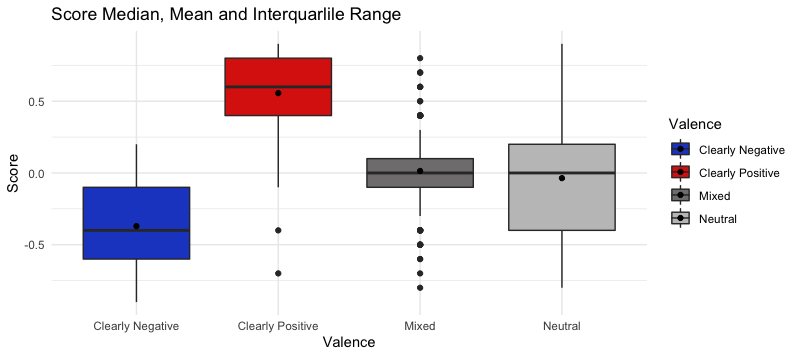


Consequently, by applying quantitative sentiment analysis with Google Cloud Natural Language API and subsequent threshold calibration for Clearly Positive and Clearly Negative responses we were able to reduce the amount of data for further qualitative analysis by 66%, from 1009 to 344 responses. More in-depth analysis of the filtered responses was carried for a dedicated article on the impact of online communications on subjective experiences under the conditions of social restrictions during COVID-19 (Froese et al., submitted)

**Quantitative analysis driven insights**

In addition to the primary filtering of records in order to reduce the number of responses for detailed analysis, we also used quantitative methods to search for insights throughout the entire corpus. After we verified the reliability of automatic coding, we applied it to all responses.

The sentiment analysis of respondents’ overall written responses is shown in **Figure 4**. Results of this analysis show a broad distribution of sentiment scores, which is consistent with the findings of other quantitative cross-cultural studies of sentiments during the pandemic (Schlegelmilch, Sharma, & Garg, 2022). This includes a few respondents with overall positive sentiment scores, but most respondents fall on the negative side of the scale. There was no difference between the distributions of overall sentiment scores between the two corpora.

**Figure 4**Score and magnitude distributions obtained from the sentiment analysis in the first (left) and second (right) corpora


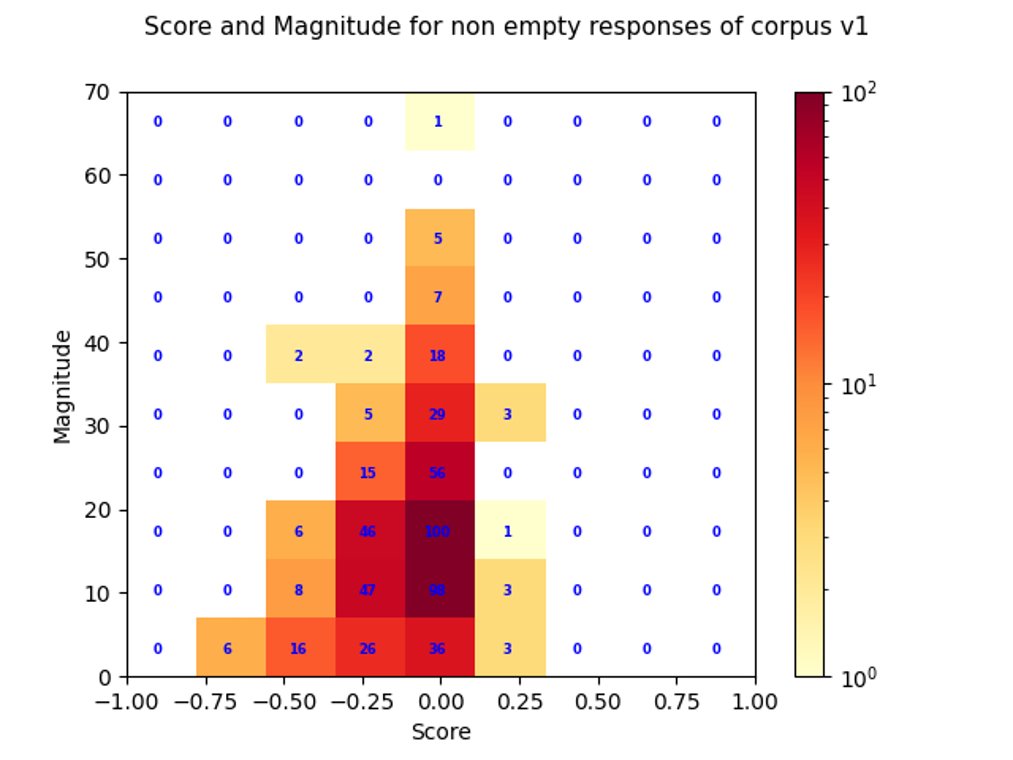

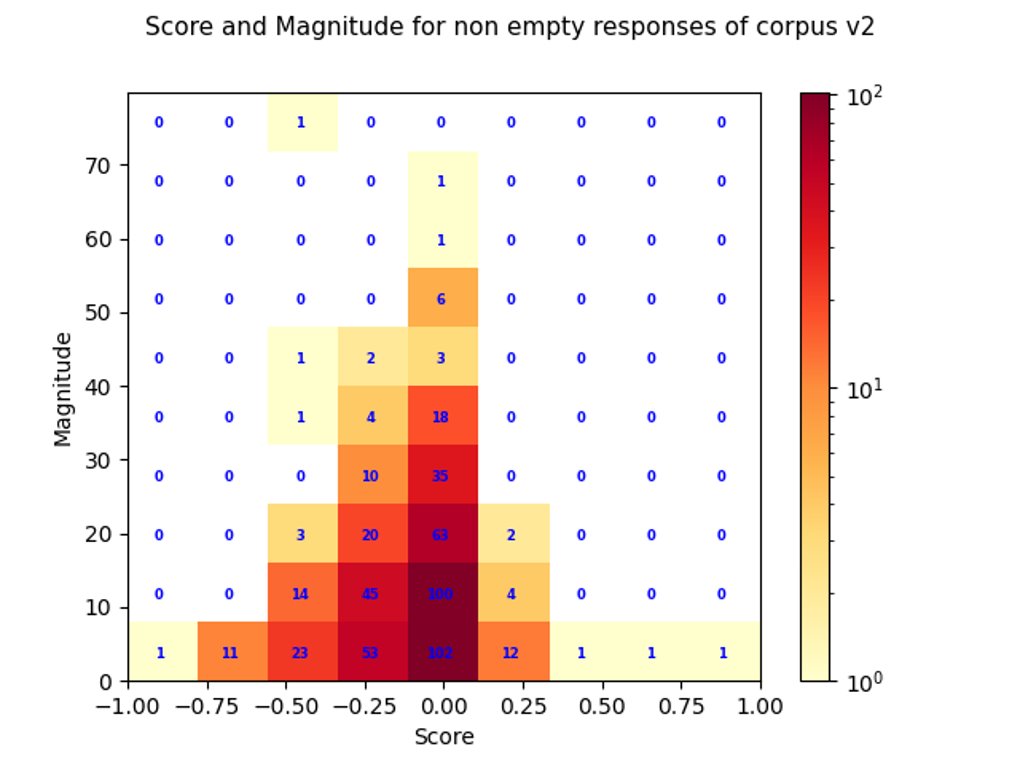


A comparison of the distribution of sentiments that is associated with each of the 31 free-text questions is shown in **Figure 5**. There were two questions that elicited a majority of positively valanced responses, namely Question 59 (“Are there things that you have been doing to better cope with the situation?”) and Question 61 (“Have you noticed any positive changes in how you experience your life, your relationships with others, or your self?”). This was the case for both corpora and is consistent with the fact that these two questions specifically asked for positive changes in people’s lived experience. In the second corpus there is an additional third question that elicited a majority of positively valanced responses, Question 43 (“How do you feel about social distancing? Do you think it is appropriate?”).

**Figure 5**: Comparison of sentiment score distributions for each question in both corpuses.

#
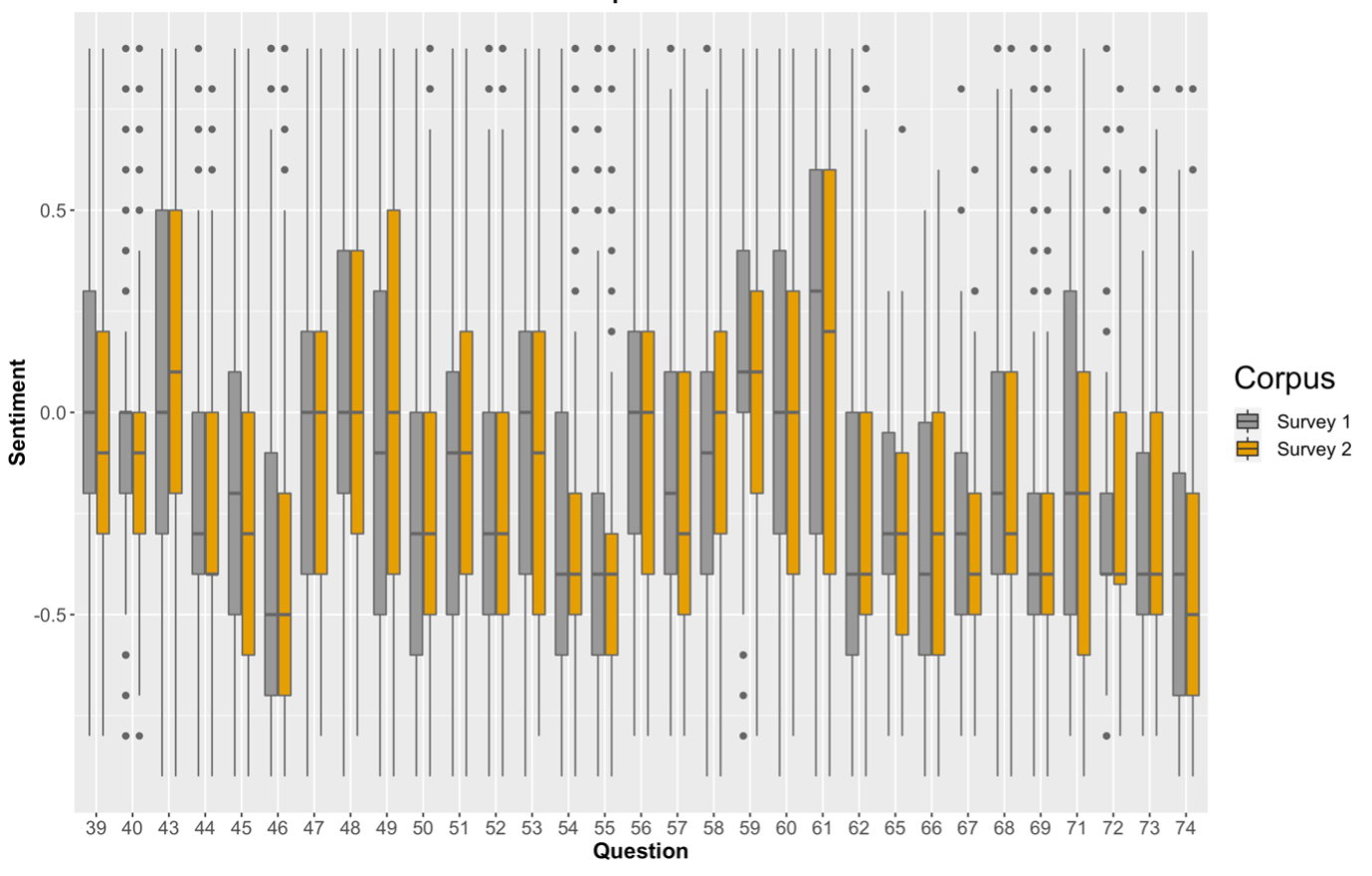


**Figures 6** and **7** show a correlation matrix for each question sentiment score against every other question sentiment score, for Corpus I and II respectively. Circles indicate correlation between different questions with the size of the circle representing the correlation strength and the color representing its direction, from negative correlation in blue to positive correlation in red. A cross indicates that a correlation is not statistically significant.

**Figure 6:** Cross-correlation matrix for sentiment scores of all questions in Corpus I.
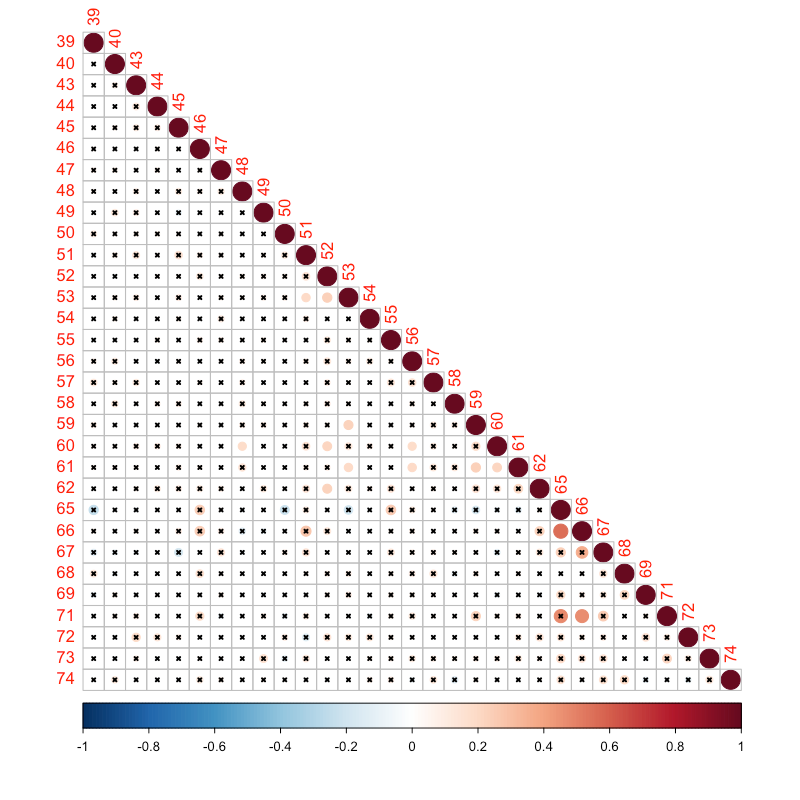


**Figure 7:** Cross-correlation matrix for sentiment scores of all questions in Corpus II.


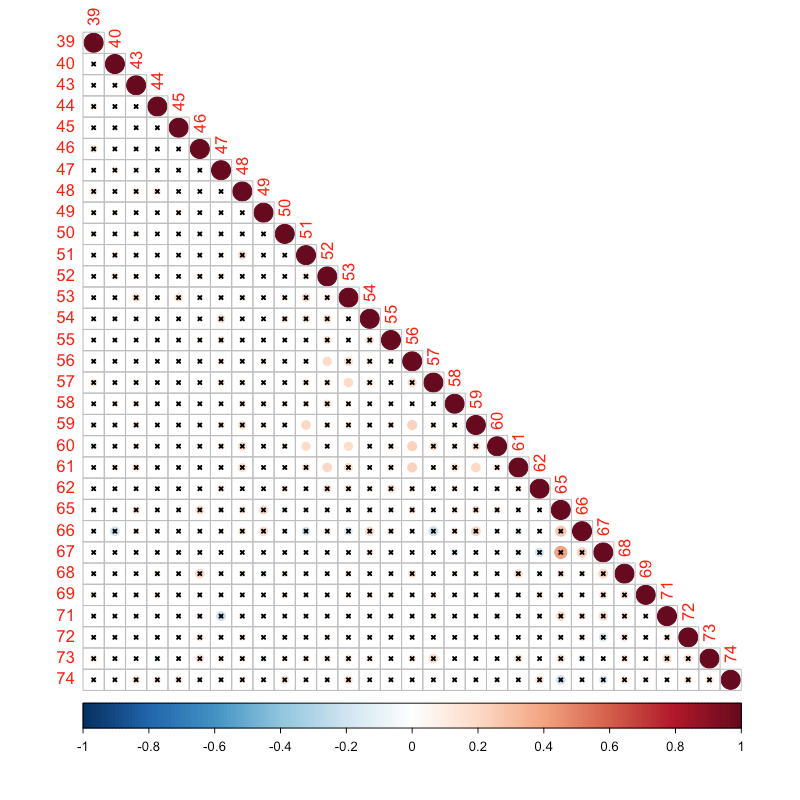


The comparison of sentiment score correlations across all 31 questions revealed that of the small subset of questions with correlated sentiment score distributions, the majority were positively rather than negatively correlated.

Regarding responses to Question 48 more specifically, in Corpus I its sentiment score was found to be positively correlated with the sentiment score of responses to Question 60 (“How has your free time changed, and how did this affect your experience of the situation?”). This finding prompted us to look deeper into the causes of this correlation. Negative valence in responses for both questions was due to two main reasons. The first is that for some people, remote working has increased their workload and reduced their free time. For some, the increase in workload is associated both with jobs and household duties. In both cases online communication technologies have not just failed to reduce the burdens of the pandemic, but have contributed to the increased stress.

**Table 3.** Examples of negatively charged responses due to the increased workload.

| **Respondent** | **Answer to Q48** | **Answer to Q 60** |
| --- | --- | --- |
| Job-related increase in workload | | |
| ES_MX_0046 | The experience was not that good. | I have more work. |
| JP_JP_0031 | I do more work-related communication via email now. I sometimes find it difficult to get my message across when communicating with students. I’m a Christian and when I led a service, I found it difficult because of the time lag. | I’ve been spending a lot of time making videos for my class, which has cut into my free time. |
| EN_UK_0099 | Yes. Technology is intermittent and a poor substitute. | Less free time as teaching remotely takes twice as long. |
| Household-related increase in workload | | |
| ES_MX_0349 | Yes it has become more frequent, but what I have noticed is indecisiveness and boredom in other people. | The time required for housework has increased, i.e. now more time has to be spent disinfecting than entering the house. This situation generates overwhelm, because before it was not done as it is done today. |
| EN_UK_0013 | It is not a great replacement to face to face interaction. Some of the emotional component missing | Very little free time. All time taken up with childcare, housework, work, with no real breaks. |
| ES_MX_0489 | Yes, but as I said, they're sort of cold, nothing like a big hug. | What free time? There isn't that anymore. I spend all my time working online, taking care of and educating my son. There are no more weekend trips to the woods, trips to the beach, to eat ice cream. I need fresh air, I need to touch the earth and get lost in the vegetation. Besides, I need the sun, my skin has become the natural tone it used to be. |
| EN_UK_0321 | Yes for church and scouting. It's difficult to interact meaningfully in a group as only one person can talk at a time and there are delays and technical hitches. In small groups where we already know each other well, it works well enough. It can be frustrating that less techy people can't mute mics and act considerately in web calls. | I have less free time as I am helping my teen son organise and keep on top of all his school work. |

The second reason for the correlation was due to the inability of people to deal with the increase in free time during the lockdown. Some respondents reported frustration and failure to manage their time wisely. Many who were comfortable with online communication technologies were able to migrate much of their lives online. However, those who were less favorable to online communication technologies often struggled to cope with the growing amount of time freed up because of the cancellation of their daily routine activities. See **Table 4** for examples.

**Table 4.** Examples of negatively charged responses due to the inability to manage free time.

| **Respondent** | **Answer to Q48** | **Answer to Q60** |
| --- | --- | --- |
| EN_UK_0198 | no i hate that sort of shit. | i had more but just wasted it as i couldn't do anything. it was like being on leave from work but having the cold so couldn't do anything. wasted relly. |
| ES_MX_0367 | I do not like video calls, I usually keep in touch through telephone calls or messages. | Well, although my free time has increased, it has been difficult to manage it and do something different. |
| EN_NA_0061 | With a video call it's fun, but in general I hate it | I have only free time, I feel useless. |
| EN_NA_0056 | Yes but found on-line communication poor alternative to direct contact | More time spent at home and socially isolated from family and friends |
| EN_UK_0492 | I hate zoom type meetings and avoid them as much as I can. Whatsap and other messaging is fine but not as good as talking. | I have so much free time that I have no direction and no drive. |
| EN_UK_0094 | Initially, yes, very much, but I am getting tired of it. Correspondence is mounting up. The technology is very helpful, though. | Working from home saves me an hour I normally spend cycling to school. The daily routine has shifted back somehow, with inner getting alter and later. This seems to be partly because my husband and I unconsciously play a game of "Who Blinks First," waiting whether the other will go and cook... I seem to spend less time on "worthwhile" things and more on mindless games and endless scrolling through social media. |
| EN_UK_0282 | I usually use online technology and have continued to do so. I used Facebook more but it became too much - people started getting irritable. Those I was friendly with retreated during later lockdown so I gave FB up. I dislike Zoom/Skype and other ways of actually seeing the other person as often the voice and picture not in synch. Nasty. | All my time is free. I've not been able to manage my time without external structure from work, meeting up with friends etc. My life seems to have had no purpose in lockdown. Could only walk and play the piano - all other things I like doing I seemed unable to do. My experience of lockdown is of being punished, tortured even. Shocked by how much I depend on other people for my well-being. We are social beings but I didn't realise how sociable I was until there was no one around. |

On the other hand, the correlation between positively charged responses indicates that for many people the negative affects of lockdowns were mitigated by the use of online tools and newly available free time. This time was often used for enjoying activities that were not given enough time before the pandemic.

**Table 5.** Examples of correlation between positively charged responses

| **Respondent** | **Score**  **(Q48)** | **Answer to Q48** | **Answer to Q60** |
| --- | --- | --- | --- |
| EN_00_0032 | 0.9 | On line has been a gift in these times and i use it frequently | Given the official recommendations I have had and continue to have much more time available to do things I’m interested in. |
| EN_00_0256 | 0.9 | I have been using on-line meeting platforms for work and social reasons - they have worked well | I had some much more free time! I relaxed, read books, watched TV and films, took solo bike rides, did tasks and chores around the house that I'd been putting off, and did some sewing and flax weaving. I also enjoyed spending time cooking and baking. I think that's why I enjoyed the isolation as much as I did. |
| EN_MX_0474 | 0.8 | Yes, it has allowed us to feel united and connected at a distance. | I have more free time, which allows me hang out more with my partner and my cat and spend more time doing what I like doing. |
| EN_UK_0062 | 0.6 | Yes, much more, via Facebook. It has been great. | My free time has expanded hugely and I have really enjoyed this. I feel much less pressured. |

It is also worth mentioning that while some people simply reported an expansion in ways of enjoying life, others were able to appreciate the causes that enabled these expansions. In quite a number of cases, it had to do with eliminating commuting time and stress and freeing up available time at home.

**Table 6.** Examples of responses pointing to decreased commuting stress.

| **Respondent** | **Score (Q48)** | **Answer to Q48** | **Answer to Q60** |
| --- | --- | --- | --- |
| EN_UK_0104 | 0.9 | Love it - I bought better tech just before lockdown - while others were panic buying toilet paper I was panic buying a good webcam, tripod, extension leads and mic! | The day isn't really broken up any more by specific events, but I am not spending any time travelling/commuting so I feel like there are more hours in the day. I was stranded in a hurricaine once with no electricity for 11 days and we went to bed at sunset and rose at dawn because we were so bored. I think having the normal home comforts makes it so much easier. I tried to set a schedule for myself but then my boyfriend moved in to shield with me and he went completely noctournal. I used to be an early riser. Now I stay up late to do online game sessions with my family who are 5 timezones earlier so I'm going to bed at 3 am around once a week. |
| EN_UK_0058 | 0.8 | Yes, I have spend a bit more time on Facebook, it's such a great way of communicating as I don't like direct face to face contact very much, as I like to have time to think about things a bit longer. Also at work, Zoom meetings instead of face to face meetings, much more relaxing. | I little more free time as I do not have to travel to work. In the beginning I had more work to do than normal but lately it's becoming a bit less. It's all been good as I'm working part time anyway. In the very beginning I felt I needed a lot of time to keep up with the news and process things though and therefore it was a bit hard to also have to work at the same time. |
| JP_JP_0273 | 0.7 | Yes, I do so definitely more frequently than before. I think it is convenient. | Since I almost always work at home, I have more leeway without spending time for commuting. I have more free time now. |
| EN_UK_0363 | 0.7 | yes. technology has been a life saver | I have had more free time than ever because I no longer travel. I have spent more time in the home. |

Although there is no significant correlation with the question specifically targeted at changes in the experience of time (“Have you noticed changes in your experience of time?”, Q57), future work could look at this from the perspective of a deeper link between technics and time (Stiegler, 1998), especially given that online communication materializes traces of our social activity in new ways, such as the preservation of our text-based conversations (Osler & Krueger, 2022), but also recordings of video chat meetings.

In Corpus II, the sentiment scores associated with Question 48 no longer reached a statistically significant correlation with the sentiment scores of any other question in the survey. It is not clear to what extent this difference compared to the first corpus reflects a change in participant’s affective experiences of technology and/or of free time, but it is consistent with the possibility that something has changed one year into the pandemic. To get a clearer insight into the concrete lived experiences of technology, and how they changed over time one should turn to a deeper phenomenological interpretation of a selection of subjective reports. We continue this work in a number of ongoing projects.

**References**

1. Froese T, James MM, Sangati F, Sangati E. From tech to tact: a corpus based study of how online communication has shaped affective experience during the COVID-19 pandemic. Phenom Cogn Sci. Under review.
2. Landis JR, Koch GG. The measurement of observer agreement for categorical data. Biometrics. 1977;33(1):159-74.
3. Osler L, Krueger J. Taking Watsuji online: betweenness and expression in online spaces. Cont Philos Rev. 2022;55(1):77-99.
4. Schlegelmilch BB, Sharma K, Garg S. Employing machine learning for capturing COVID-19 consumer sentiments from six countries: a methodological illustration. Int Market Rev. 2022.
5. Stiegler B, Beardsworth R, Collins G. Technics and time, 1 : the fault of Epimetheus. Stanford, California: Stanford University Press; 1998. xv, 295 p.
